# Probiotics: A Promising Candidate for Management of Colorectal Cancer

**DOI:** 10.3390/cancers13133178

**Published:** 2021-06-25

**Authors:** Ashutosh Tripathy, Jayalaxmi Dash, Sudhakar Kancharla, Prachetha Kolli, Deviyani Mahajan, Shantibhusan Senapati, Manoj Kumar Jena

**Affiliations:** 1 Department of Biotechnology, School of Bioengineering and Biosciences, Lovely Professional University, Phagwara, Punjab 144411, India; ashutosh.11701104@lpu.in (A.T.); deviyani.11919673@lpu.in (D.M.); 2 Tumor Microenvironment and Animal Models Laboratory, Institute of Life Sciences, Bhubaneswar, Odisha 751023, India; jayalaxmi@ils.res.in (J.D.); senapati@ils.res.in (S.S.); 3 Devansh Lab Werks, 234 Aquarius Drive, Homewood, AL 35209, USA; sudhakar@devlabwerks.com; 4 Microgen Health Inc., 14225 Sullyfield Cir Suite E, Chantilly, VA 20151, USA; Prachetha@microgenhealth.com

**Keywords:** probiotics, colorectal cancer, inflammatory bowel disease, immunomodulation, intestinal barrier function, apoptosis, eubiosis

## Abstract

**Simple Summary:**

Colorectal cancer being the third most frequently diagnosed cancer type, is creating enormous physical, financial, and emotional burden on individuals as well as on the health care system. Probiotics have been there in the limelight due to their numerous health benefits. In recent decades use of probiotics for the management of colorectal cancer is becoming increasingly popular owing to their positive and favourable outcomes in many in vitro, in vivo, and clinical investigations. The positive results are believed to be the manifestation of multiple beneficial effects exerted by probiotics acting constitutively. This review provides an overview of several mechanisms of probiotic activity supported by evidences from in vitro and in vivo investigations, establishing the promising role of probiotics as a candidate for colorectal cancer management.

**Abstract:**

Colorectal cancer (CRC) is the World’s third most frequently diagnosed cancer type. It accounted for about 9.4% mortality out of the total incidences of cancer in the year 2020. According to estimated facts by World Health Organization (WHO), by 2030, 27 million new CRC cases, 17 million deaths, and around 75 million people living with the disease will appear. The facts and evidence that establish a link between the intestinal microflora and the occurrence of CRC are quite intuitive. Current shortcomings of chemo- and radiotherapies and the unavailability of appropriate treatment strategies for CRC are becoming the driving force to search for an alternative approach for the prevention, therapy, and management of CRC. Probiotics have been used for a long time due to their beneficial health effects, and now, it has become a popular candidate for the preventive and therapeutic treatment of CRC. The probiotics adopt different strategies such as the improvement of the intestinal barrier function, balancing of natural gut microflora, secretion of anticancer compounds, and degradation of carcinogenic compounds, which are useful in the prophylactic treatment of CRC. The pro-apoptotic ability of probiotics against cancerous cells makes them a potential therapeutic candidate against cancer diseases. Moreover, the immunomodulatory properties of probiotics have created interest among researchers to explore the therapeutic strategy by activating the immune system against cancerous cells. The present review discusses in detail different strategies and mechanisms of probiotics towards the prevention and treatment of CRC.

## 1. Introduction

Cancer is one of the leading causes of death worldwide, and the number of expected cases is going to rise to 29.5 million by 2040 [1]. Maintaining a balanced and healthy diet and physical activity can prevent the occurrence of cancer [2]. Owing to the excessive use of tobacco, alcohol consumption, unhealthy diet, and physical inactivity, the number of cases is increasing day by day [3].

Current statistical data from “CANCER TODAY (WHO)” shows that colorectal cancer (CRC) is the third most diagnosed cancer type worldwide, accounting for about 1,931,590 (10% of total cancer cases) of new cases in the year 2020. The mortality rate of CRC was 935,173 (9.4% of total mortality in cancer cases) in the year 2020, which is a major concern in the health sector. The incidences of CRC are found to be more prevalent in the European, North American, and Australian continents than in Asiatic countries [4]. The aetiology suggests both genetic as well as environmental factors contributing to a set of conditions that lead to CRC in many people. The major risk factors that are involved in this cancer type include age, lifestyle-related factors such as diet, physical activity, obesity, smoking, and alcohol consumption, and previous family history of adenomatous polyps/Inflammatory Bowel Disease (IBD)/Colorectal cancer, etc. Inflammatory Bowel Disease (IBD) occurs due to prolonged inflammation and increases the risk of CRC [5]. With limited evidence, it is believed that inflammation leads to altered mucosal glycosylation and an increase in arachidonic acid metabolism, which promotes the development of colorectal cancer [6]. The close relationship between diet and CRC is an established fact, and the incidence of CRC can be significantly reduced (up to 70%) by consumption of a healthy and balanced diet [7,8]. The microbiota composition changes with a change in diet, leading to a change in overall health pattern. Consumption of diet with high animal fat, red meat increases the risk of CRC, which is driven by secretion of N-nitroso compounds, which are carcinogenic, by gut microflora [9,10,11]. It is evident that the western lifestyle, which involves red meat as a large part of their diet habits, is one of the major causes of the occurrence of CRC [12].

Thanks to the recent advances in early detection and diagnosis of CRC, which involve the use of biomarkers to detect mutations and gene expression profiling, there are around 90% cases with a 5-year survival rate having a localized stage of colorectal cancer. At the same instance, colorectal cancer-related morbidity in young adults and chemoresistance to existing therapies remains a major challenge. Although screening at an early stage can significantly improve survival, most of the patients with colorectal cancer are diagnosed at an advanced stage. Neoadjuvant therapy before surgery, which is followed by chemotherapy, is recommended for such patients. However, pharmacologic therapy often is associated with toxic and harmful side effects, and patients eventually develop chemoresistance. The conventional treatment strategies for CRC mainly involve surgery, radiation therapy, and chemotherapy, but these therapies have limitations due to the non-discrimination between normal and cancerous cells and induce cancer in surrounding tissues due to exposure to radiation. Surgery and polypectomy are limited to small and localized tumors and create post-operative complications. A more recent therapeutic approach includes immunotherapy which is only used as a supplement therapy and is often insufficient to completely cure cancer disease [13,14]. Therefore, there is a need for some alternate approaches to prevent colon cancer, and researchers all around the globe are trying to focus on finding out new interventions for the cure of CRC. Among the regulatory factors that cause CRC the diet, lack of exercise, and lifestyle are measure. So, suggestions to prevent the occurrence of colon cancer include an amendment in lifestyle, consuming more vegetable-based and fiber-rich diets, and most importantly, switching to functional foods.

Functional foods are known to have additional health benefits and disease prevention apart from nutritional values. They may be similar to conventional food and consumed as a regular diet but have physiological benefits reducing the risk of chronic diseases. Probiotics, which have been used since ancient times in fermented food and beverages, have now been redefined by FAO/WHO as live microorganisms that, when administered in adequate numbers, provide health benefits to the host [15]. Though probiotics were very well included in the diet for their role in the improvement of digestive functions, with the progression of time, it has now been proved important in physiological and immunological effects in the host. They naturally inhabit the human gut and are also supplied exogenously for their beneficial role in a mutualistic environment with other gut microbiota. The probiotics interact with the host and resident microflora in the intestinal niche giving rise to numerous host-microbe interactions that are being beneficial for both the host as well as the microbes. Our intestine, especially the colon and the rectum, harbour the largest and diverse range of microbial communities, which maintain homeostasis by constant crosstalk with mucosal immune cells and intestinal epithelial cells. The factors, including nutrition, stress condition, lack of physical activity, alcohol consumption, and prolonged medications, often create an imbalance in intestinal microflora and inflammation, leading to inflammatory bowel disease; however, the long-term acute inflammation may lead to the development of cancer in the colon. The probiotics are known to have an immunomodulatory role and different health benefits, which can be used in the prevention and treatment of CRC, restore the dysbiotic intestinal microflora, and work in coordination with immune cells to fight against the disease. The probiotics adopt different pathways to protect the colon epithelial cells from cancer disease. The first evidence of preventive effects of the probiotic *Lactobacillus acidophilus* was reported in the year 1980, using male inbred F344 rats that were induced with 1,2-dimethylhydrazine dihydrochloride (DMH), causing colorectal cancer [16]. Since then, many in vitro and in vivo studies have provided strong evidence for the use of probiotics in the prevention and treatment of different types of cancers. 

**Figure 1:**
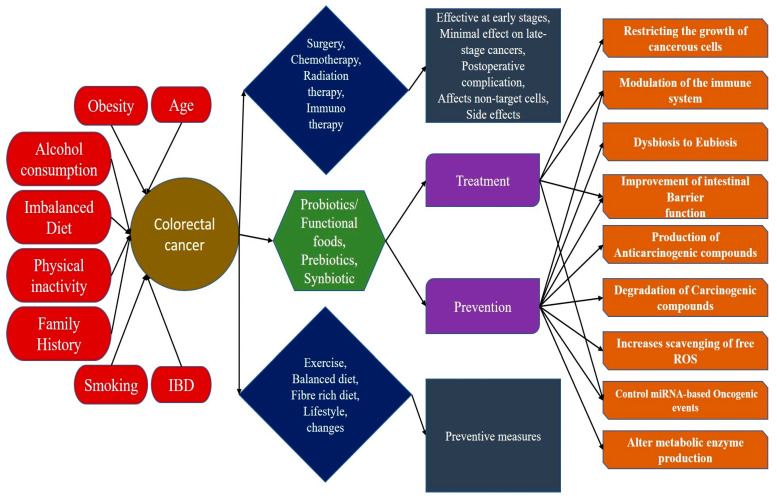
An illustration of the holistic picture of different causes and treatment strategies for colorectal cancer. Different environmental and lifestyle factors contribute to the development of colorectal cancer. Treatment strategies for colorectal cancer come with some additional drawbacks, such as its effectivity at only early stages, minimal effect on late-stage cancer, off-target effects and complications. A change in lifestyle decreases the chances of the occurrence of the disease. Probiotics/functional foods provide preventive measures through a range of different mechanisms and hold the ability for becoming a possible treatment strategy for colorectal cancer due to its anticancer and immunomodulatory properties.

Probiotics can confer preventive actions against CRC through a range of mechanisms [17,18]. Most of the time the mechanisms work constitutively, exerting positive effects on the intestinal microbiota and preventing the colon and rectum from the environmental stress factors which often induce carcinogenesis. The primary mechanisms include the establishment of eubiosis condition, improvement of the intestinal barrier function, modulation of the intestinal immune system, production of anticarcinogenic compounds, and degradation of the carcinogenic compounds in the intestinal milieu. These organisms also induce pro-apoptotic and antiproliferative effects on the cancerous cells, which suggests their possible implications in the treatment of CRC. They can also alter the secretion of different metabolic enzymes (which convert precursor compounds into carcinogenic compounds) by mutual interaction with other intestinal microbes. This review article discusses in detail about the different mechanisms adopted by probiotics that can prevent as well as aid other treatment strategies for the management of CRC. A complete idea of the cause, treatment strategies for colorectal cancer along with the role of probiotics for both prevention and treatment of cancer with an array of mechanisms is demonstrated in Figure 1.

## 2. Mechanism of Action of Probiotics

### 2.1. Restricting the Growth of Cancerous Cells

The probiotics can restrict the growth of cancerous cells by inducing apoptotic pathways (both intrinsic and extrinsic pathways). The pro-apoptotic effect is confirmed in many in vitro experiments, which are generally accompanied by expression or suppression of apoptosis-related proteins such as death-ligand receptors, pro-caspase, caspase-3, -8, and -9, Bax/Bak, Bcl-2, and Bcl-x proteins [19]. They also put a check on cell cycle growth phases to restrict their differentiation and proliferation, which can be marked by a change in cyclin expression. It is observed that two probiotic species, *Propionibacterium acidipropionici* and *Propionibacterium freudenreichii*, secret produce Short-chain fatty acids (SCFAs) such as propionate and acetate, which induce cellular apoptosis in human colon cancer cell line HT-29 and human colorectal adenocarcinoma or Caco-2 cells. Cellular apoptosis is induced through the activation of the caspase 3 enzyme, followed by chromatin condensation, apoptotic nuclei body production, and generation of reactive oxygen species [20]. Regular consumption of dietary fibres indirectly increases the SCFA production via modulation of gut flora. The probiotic bacteria *Lactobacillus rhamnosus* GG (LGG) and *Bifidobacterium lactis* Bb12, when added as inoculum along with fecal slurries for the fermentation of wheat aleurone, enhanced the fermentation efficiency. The fermentation supernatant was characterized by an increased concentration of SCFAs such as butyrate, acetate, and propionate. The butyrate not only serves as a major energy source for colonocytes and enhances their survivability but also causes inhibition of neoplastic cells inducing apoptosis. These SCFAs were found to inhibit the growth of LT97 colon adenoma cells and arrest the cell cycle in HT-29 cells. The cell cycle arrest in HT-29 colon cancer cells was marked by the check on G0/G1, modulation of p21 and WNT2B mRNA expression, and modulation of differentiation activity, which led to apoptosis of the cells [21]. Additionally, four probiotic strains, such as *Pediococcuspentosaceus* FP3, *Lactobacillus salivarius* FP25, *L. Salivarius* FP35, and *Enterococcus faecium* FP51, isolated from healthy infant faeces, showed an antiproliferative effect on Caco-2 cells, which is driven by their ability of adherence to cancer cell lines and production of SCFA such as butyric and propionic acids. The probiotic properties of these strains were validated by assessing their tolerance to extreme conditions of pH 2.5 and 0.3% bile salt of the gastrointestinal tract (GIT), absence of hemolytic effect on blood agar, and antagonistic effects against foodborne pathogens [22]. 

*Propionibacterium freudenreichii* secretes SCFAs such as propionate and acetate, which can kill the HT-29 cells through activation of the mitochondrial apoptosis pathway. It is found that the supernatant and ultrafiltrate of milk fermented by *Propionibacterium freudenreichii*, caused apoptosis in HT-29 cells mediated by both the extrinsic and intrinsic death pathways. The synergistic effect of both pathways was marked by increased expression of TNF-Related Apoptosis-Inducing Ligands (TRAILs) (TRAIL-R1/DR4, TRAIL-R2/DR5), activation of Caspase-3, -8, -9, and inhibition of Bcl-2 expression [23]. Another Lactic acid bacteria (LAB), *Lactobacillus casei* ATCC 393, had shown an antiproliferative as well as a pro-apoptotic effect on CT26 (Murine) and HT-29 (Human) colon carcinoma cell lines by upregulation of TRAIL and downregulation of cyclin D1 and Baculoviral IAP Repeat Containing 5 (BIRC5a) gene. The cell viability in these cell lines depends on the bacterial concentration and co-incubation time. A 78% and 52% decrease in viability were seen for HT-29 and CT26, respectively, at a bacterial concentration of 10^9^ CFU/mL with a co-incubation time of 24 h [24]. Similarly, heat-killed cells and cell-free supernatant (CFS) of *Lactobacillus plantarum* A7 and commercial probiotic strain *Lactobacillusrhamnosus* GG had also shown an antiproliferative effect on Caco-2 and HT-29 cancerous cells, which is characterized by organic acid production capacities of Lactobacilli genera. The results have shown that CFS of *Lactobacillus plantarum* A7(not established as probiotic) is more effective in inhibiting the growth of cancerous cells than the established probiotic strain (LGG). This study indicates that LAB not fulfilling all the probiotics attributes but only able to produce organic acids could exhibit the antiproliferative effect [25]. The supernatants collected from the cultures of *Lactobacillus casei* UT1 had shown a pro-apoptotic effect on human colon cancer cells HCT116 in a time and dose-dependent manner, which is marked by an increase in the population of colon cancer cells in sub G1 phase [26].

The antiproliferative activity on Caco-2 cell lines was observed by culture media and whole live cells of probiotic strains *Enterococcus faecium* RM11 and *Lactobacillus fermentum* RM28. These strains have shown a better survival rate in the gastrointestinal tract model (pH 2.5 and 0.3% Bile salt) and adherence with Caco-2 cell lines, qualifying them to be potential probiotic candidates for colorectal cancer treatment [27]. The ErbB receptor family (a group of receptor tyrosine kinase) is an epidermal growth factor receptor family that includes and is found to be over-expressed in different types of cancers [28]. It is also evident that EGF (Epidermal growth factor) or ErbB-mediated signaling has a major role in the proliferation of tumor cells [29]. The conditioned culture medium of a probiotic bacterium *Bacillus polyfermenticus* had shown inhibition of growth of HT-29, colorectal adenocarcinoma cells DLD-1, and Caco-2 cells. Experimental evidence suggests that inhibiting properties of a probiotic bacterium are mediated through the inhibition of the ErbB receptor-dependent pathway with the suppression of ErbB2 and ErbB3 proteins [30]. An investigation on the probiotic bacteria *Lactobacillus pentosus* B281 and *Lactobacillus plantarum* B282 revealed that they could adhere to Caco-2 cells effectively. The conditioned cell-free media showed the antiproliferative effect on Caco-2 cells, directed by an arrest of the G1-phase of the cell cycle through downregulation of cyclins A, B1, B2, and E proteins. Moreover, it was found that the bioactive compounds causing the antiproliferation effect are thermostable in nature [31]. Similarly, cell extract from *Lactobacillus acidophilus* LA102 and *Lactobacillus casei* LC232 bacteria showed antiproliferative and cytotoxic activity on Caco-2 (37% and 48%) and colorectal adenocarcinoma cells HRT-18 (68% and 45%), respectively, without showing any detrimental effect on normal Vero cells (African green monkey kidney cells). The inhibitory concentration (IC50) values were 1.60 and 15.4 by LA102 and LC232 strains, respectively, for Caco-2 cells; and 2.50 and 6.20 by LA102 and LC232 strains for HRT-18 cells, respectively [32]. Another study using supernatant from *Lactobacillus acidophilus* (ATCC 4356) and *Lactobacillus casei* (ATCC 39392) showed an antiproliferative and apoptotic effect on Caco-2 cell lines, while the cell extracts from both bacterial strains showed antiproliferation, apoptotic, and necrosis effect. Both cell lysate and supernatant showed a negative effect on the invasion and migration of cancer cells [33], suggesting cell lysate could be used to prevent metastasis of cancer cells in later stages. Investigating the effect of cell-bound exopolysaccharides (cb-EPS) isolated from *Lactobacillus acidophilus* 606 on HT-29 cell line showed promising results of antitumorigenic effect. This effect is driven by the autophagic cell death induced by the Beclin-1 regulatory molecule, which is an autophagy protein. The antitumorigenic effect is also indirectly showed by GRP78 protein, which regulates the cell autophagy by ER stress (Endoplasmic reticulum stress) along with the crosstalks of apoptosis-inducing factors such as Bcl-2 and Bak proteins [34]. A similar study showed that crude exopolysaccharide produced by *Lactobacillus plantarum*-12 showed an antiproliferative effect on HT-29 cells, which is marked by the expression of proliferating cell nuclear antigen (PCNA). The pro-apoptotic effect was marked by the increased expression of apoptotic proteins such as Bax, Cytochrome C, caspase-3, -8, and -9 enzymes and decreased expression of the antiapoptotic protein Bcl-2 in the HT-29 cells [35]. 

Both pathogens and commensals are inhabiting together gut environment though the commensals outgrow the pathogens in many folds. A differential apoptosis induction pattern on the Caco-2 cell line by pathogenic and commensal *Escherichia coli* strain, probiotic bacteria, and a gut bacterium *Atopobiumminutum* was demonstrated by Altonsy et al. The findings demonstrated a strong apoptotic effect by pathogenic *E. coli* (Enteropathogenic (EPEC) 086 NCTC 8621 and Verocytotoxin VTEC Vt−NCTC 12900), mild apoptotic effect by probiotic bacteria (*Lactobacillus rhamnosu*s GG, *Bifidobacterium latis* Bb12), and gut bacterium (*Atopobiumminutum* X67148), and no apoptotic effect by commensal *E. coli* K-12 strain. This study revealed that the apoptosis of the Caco-2 cancerous cells was driven by the mitochondrial pathways with the release of cytochrome c, BAX translocation, and activation of caspase-8 and caspase-3 enzymes [36]. Warburg effect is a metabolic rewiring process adopted by tumor or cancerous cells to increase the uptake of glucose and convert them to lactate irrespective of the presence of oxygen [37]. This helps the cancerous cells to maintain active growth and proliferation and ensure their long-term survival in the tumor microenvironment. In another study, the microorganism *Streptococcus thermophilus* (19258) showed apoptosis, antiproliferation, and cell cycle arrest effect on HCT116, HT-29, and Caco-2 cancerous cells. These effects are found to be driven by the production of galactose (which is produced primarily by the probiotic bacteria), followed by interfering with energy homeostasis and establishing an anti-Warburg effect phenotype [38].

In recent studies, the adverse effect of probiotics was reported in immune-compromised patients. The pro-apoptotic effect of heat-inactivated probiotic yeast strain *Saccharomyces cerevisiae* PTCC 5052 on SW-480(human colorectal adenocarcinoma cells) was noticed in a study. This effect was facilitated by the modulation of the Akt/NF-κB signaling pathway followed by apoptosis marked by the upregulation of BAX, cleaved caspase-3, and cleaved caspase-9 and downregulation of Bcl-XL, procaspase-3, and procaspase-9, p-Akt1, Rel-A expression [39]. A similar study demonstrated that both viable and heat-killed strains of *Lactobacillus paracasei* IMPC2.1 and *Lactobacillus rhamnosus* ATCC 53,103 (LGG) showing the growth inhibition and apoptotic effect on the DLD-1 colorectal cancer cell line [40]. This study suggests that inactivated probiotics can be used as an alternative to live bacteria in immune-compromised patients.

To overcome the limitation of conventional chemotherapeutic drugs, especially in metastatic colorectal cancer (CRC), Baldwin et al. demonstrated the use of *Lactobacillus acidophilus* CL1285 and *Lactobacillus casei* LBC80R as an adjuvant with chemotherapeutic drug 5-fluorouracil (5-FU) to increase the sensitivity of LS513 colorectal cancer cells towards the drug. Results showed a 40% increase in apoptotic activity by 5-FU (100 μg/mL) when mixed with a mix of live lactic acid bacteria (LAB) (10^8^ CFU/mL) and incubated with LS513 cell line for 48 h. It is also demonstrated that irradiation-inactivated LAB also showed the same level of apoptotic activity in contrast to microwave-inactivated LAB, which showed reduced apoptotic activity. An increased rate of Caspase-3 activation and downregulation of p21 protein is a possible mechanism of the synergistic effect of 5-FU and LAB [41]. Another study also confirms the *Lactobacillus plantarum* (CCARM 0067) supernatant enhance the effect on 5-FU-resistant HT-29 and HCT116 cells by induction of caspase-3 activity and inactivation of Wnt/β-catenin pathway, which is marked by the decreased expression of cancer stem cell markers CD44, CD133, CD166, and ALDH1 [42]. This study suggests the use of both bacterial cells and cultured supernatant as an adjuvant with chemotherapeutic drugs to increase the chemosensitivity of cancerous cells. 

Table 1 shows the list of probiotic bacteria that have significant pro-apoptotic effects, antiproliferation, and cell cycle arrest effects on different cancerous cell lines.

### 2.2. Modulation of the Immune System

The association and communication of gut microbiota and intestinal immune system are very important in creating homeostasis conditions. The intestinal microbiome train immune cells to behave optimally to eliminate the pathogenic microbes as well as to show tolerance to commensal microbes. Toll-like receptors (TLRs) present on epithelial cells often respond to different microbial-derived factors, which act as ligands/Microbes-associated molecular patterns (MAMPs) and initiate a cascade of immunological responses. Dysbiosis (an imbalanced gut microflora ecosystem) conditions lead to activation of MAPK (mitogen-activated protein kinases) pathway and nuclear translocation of NF-κB that leads to the secretion of different pro-inflammatory cytokines such as IL-8 and nitric oxide, which consequently leads to the occurrence of IBD and colorectal cancer. Administration of probiotics re-establishes the eubiosis (a balanced gut microflora ecosystem) conditions and leads to the secretion of anti-inflammatory cytokines such as IL-10 and TGF-β2 by regulatory T (T_reg_) cells. The T_reg_ cells are the differentiated T cells, which play a role in the suppression of the immune system so that it will not react to self-antigens and antigens of commensal microbes to promote their tolerance. Both commensal and harmful microbes are recognized and presented by the antigen-presenting cells such as dendritic cells (DCs), which travel to the mesenchymal lymph node and can direct the differentiation and maturation of T-cells into T_reg_, Th1, Th2, and Th17 cells. Subsequently, these differentiated Th cells secrete various pro-inflammatory cytokines such as interleukin-1 (IL-1), IL-12, and IL-18, tumor necrosis factor-alpha (TNF-α), interferon-gamma (IFNγ), and T_reg_ secretes different anti-inflammatory cytokines, such as IL-10 and TGf-β2, and regulates the inflammation and homeostasis in the intestine [60,61,62].

In vitro and in vivo studies proved chemokine IL-8 is overexpressed in CRC cells and has tumor-promoting and pro-angiogenic effects along with the increase in metastatic and chemoresistance property suggesting, IL-8 to be a potential target for colorectal cancer therapy [63]. In a motive to provide a safe effective alternative to live bacteria, Lopez et al. experimentally confirmed that both live *Lactobacillus Rhamnosus* GG (LGG) and UV-inactivated LGG downregulated the flagellin induced IL-8 expression in Caco-2 cells by 66% and 59%, respectively. In the cytoplasm, the NFκB(nuclear transcription factor) is bound to inhibitor molecule IκB that restricts the NFκB nuclear translocation. The gut commensal bacteria inhibit the degradation of IκB, hence nuclear translocation of NFκB, thus regulating the inflammatory response in the gut through the secretion of chemokines such as IL-8 [54].

Different colon cancer cell lines such as DLD-1, HT-29, and LoVo cells were treated with probiotic strain *Lactococcus lactis* NK34. The findings revealed the reduction of proliferation of these cancerous cells, confirming the cytotoxicity effect of the probiotics. There was also an immunomodulatory effect of these probiotic bacteria on RAW 264.7 macrophage cells, marked by a reduction in pro-inflammatory cytokine and nitric oxide (NO)production [55]. Similarly, the microorganism *Bacillus polyfermenticus* KU3, isolated from a Korean dish, shows probiotic properties and has an antiproliferative effect on LoVo and HT-29 cells with a reduction in the production of pro-inflammatory cytokines COX-2 and TNF-α, and nitric oxide [56]. 

A probiotic cocktail made up of *B*. *longum*, *B*. *bifidum*, *L*. *acidophilus*, *L*. *Plantarum* with prebiotics as resistant dextrin, isomaltooligosaccharides, fructose oligosaccharides, and stachyose showed antiproliferative effect accompanied by reducing the metastatic properties such as migration and invasion in CT26 cells. The antitumorigenic property of these synbiotics driven by the T-cell-mediated immune response marked by an increase in CD8+ T cells [57]. It is found that the growth inhibition potential of cell-free supernatant (CFS) isolated from *Bifidobacterium adolescentis* SPM0212 on HT-29, SW-480, and Caco-2 cancerous cell lines work in a dose-dependent manner. The isolated CFS also induced the production of TNF-α from the macrophage cell line (RAW-264.7), which has a prominent immunomodulation role in tumor cell inhibition [58]. Conditioned medium prepared from *Clostridium butyricum* (ATCC 19398) and *Bacillus subtilis* (ATCC 23857) has shown apoptotic and cell arrest effect on HCT116 (Human colon cancer cell line) and SW-1116 (Human colorectal adenocarcinoma) cells. It is found to be driven by lowering inflammation marked by decreased expression of TLR4 and NF-κB. The immunomodulatory effect was achieved by modulation of Th17 cells. Furthermore, the antiproliferation effect is marked by a decreased expression of p-ERK and increased expression of P21 [59]. Multiple studies have provided empirical evidence that shows the direct and/or indirect effect of probiotics on the better outcome of immunotherapy. Lysates of L acidophilus with CTLA-4 blocking antibodies have shown enhanced antitumor effect in a mouse colon cancer model [64]. (The immunomodulatory effect of different probiotics is compiled in Table 1.).

### 2.3. Dysbiosis to Eubiosis

Eubiosis is defined as the condition of a balanced and homeostatic gut microflora ecosystem. On the contrary term, “Dysbiosis” is often referred to as the disruption of homeostatic conditions present in the intestine and the commensal intestinal microflora and are characterized by a significant loss in population of beneficial microorganisms, the prevalence of harmful microorganisms, or depletion in microbial diversity [65]. Establishing a eubiosis condition could help in the prevention and treatment of colorectal cancer. Rebalancing the gut ecosystem can be accomplished by administration of probiotics, prebiotics, synbiotics, which can create homeostasis by neutralizing harmful pathogens, helping the growth of indigenous beneficial bacteria, modulating the immunological responses, and repairing intestinal mucosa [66]. The composition of intestinal microflora is very crucial and influences the response to treatments of colorectal cancer [67]. Variation in the local intestinal environment, which involves uptake of different nutrients, administration of drugs, immunological responses, and alterations in intestinal mucosa, can create an imbalance in natural microbial phyla, resulting in increased colonization of harmful and pathogenic strains. The imbalance in microbial phyla is often seen to be escalated by leukocyte-driven oxidative stress, secretion of bacteriocins by harmful bacteria, and prevalence in the population of bacteriophages [68]. Song et al. reviewed the close relationship between the occurrence of colorectal cancer with environmental factors, diet, and gut microbiome composition [69]. Gut microbiota profile of healthy host composed of mainly Firmicutes, Actinobacteria, Bacteroidetes, and proteobacteria phylum. Firmicutes are composed of major orders such as Clostridiales, Bacteroidales, Bifidobacteriales, Enterobacterales, and Lactobacillales, and it is found that *Bifidobacterium longum* is the most abundant species and belongs to the family of Bifidobacteriaceae [70].

A significant difference can be seen in the microflora composition of CRC patients with respect to healthy individuals [71,72,73]. A decrease in butyrate-producing bacteria belongs to a family of Lachnospiraceae, and the genus of *Roseburia* has been seen in CRC patients. Bacteria belong to the genus of *Enterococcus, Escherichia*/*Shigella*, *Klebsiella*, *Streptococcus,* and *Peptostreptococcus* are found to be more prevalent in CRC patients whereas, on the other hand, healthy individuals have guts enriched with bacteria related to * Bacteroides vulgates* and *Bacteroides uniformis* species [74]. Crypts are intestinal glands and are often named colonic crypts. Even a variation in microbial communities is found in the right and left crypt of CRC patients. Left-crypt (crypts of transverse and descending colons) was found to be populated by *Parvimonas micra,* whereas right-crypt (crypts of the caecum and ascending colon) was populated by *Fusobacterium periodonticum* and *Bacteroides fragilis* [75]. A gene profile meta-analysis found the prevalence of invasive bacterial biofilm, tumorigenic symbiont *Bacteroides fragilis*, and oral pathogens such as *Fusobacterium nucleatum*, *Parvimonas micra*, and *Peptostreptococcus stomatis* [76]. Metagenomes with a prevalence of protein genes and mucin catabolism increased the production of bile acids from metagenomes of CRC patients, and depletion in carbohydrate degradation genes are characterized as unique microbial signatures specific for colorectal cancer [77].

Some alternate strategies include Fecal Microbiota Transplantation (FMT) and bacterial consortium transplantation, and less explored methods, such as phage therapy and predatory bacteria-based strategies, can also be implemented for the establishment of eubiosis conditions [78]. A commercial probiotic cocktail VSL#3, which consists of eight probiotic species [79], showed a remission rate of 77% in patients suffering from mild to moderate ulcerative colitis without any adverse effect [80], and remission was also seen in children suffering from Active ulcerative colitis when treated with VSL#3 and IBD therapy [81].

### 2.4. Improvement of Intestinal Barrier

Our intestinal epithelial cells guard the internal environment against pathogenic bacteria, toxic substances, and stress factors. Their paracellular (passing through space in between two cells) and trans-cellular (passing through the cell) permeability properties regulate the movement of water, ions, and nutrients as well as restrict the movement of harmful entities. The barrier function is made up of three components known as adherins junctions (AJs), tight junctions (TJs), and desmosomes. AJs comprise the interaction of complexes such as cadherin-catenin and Nectin-afadin with the cytoskeletal elements of intestinal cells. TJs are present at the intersection of apical and lateral membranes and can selectively control paracellular permeability. They are made up of transmembrane proteins such as occludin, claudins, and junctional adhesion molecules (JAMs), which are intracellularly connected to cytoskeletal entities by Zonula occludens-1/2/3, PDZ domain-containing proteins, and cingulin. The intestinal barrier function is modulated by immune cells such as mast cells as well as cytokines such as IFN-γ, TNF-α, IL-4, and IL-13 and can be disrupted by pathogenic organisms such as *Vibrio cholera*, *Enteropathogenic E. coli*, *Clostridium perfringens*, and their toxins. It is observed that alcohol consumption and non-steroidal anti-inflammatory drugs can exert significant damage on barrier functions [82]. Evidence is there for disruption of the TJs, which compromise the integrity of barrier function by enteric pathogens and their toxins in chickens [83]. The disruption of tight junction protein complexes or dysregulation of paracellular permeability leads to the development of Inflammatory bowel diseases and is often accompanied by IBD-associated CRC [84]. Cancer metastasis and tumor invasion are accelerated when there is a disruption of TJs, which leads to increased permeability of the paracellular route, and modulation of TJs is found to be an essential marker of metastasis events [85].

Probiotics secrete Short-chain fatty acids (SCFA), and these SCFAs have shown positive results in improving the intestinal barrier function. It is found that SCFAs such as butyrate, propionate, and acetate have shown a protective effect against the disruption of barrier function induced by ethanol [86]. Ethanol cause disruption in TJs and epithelial cytoskeletons and increased metabolic stress. SCFAs decrease the metabolic stress and reinforce the TJs by activating AMP-activated protein kinase (AMPK) in Caco-2cells [87]. Butyrate derived from bacterial sources stabilized the Hypoxia-inducible factor (HIF) by decreasing O_2_ concentration and improve the epithelial barrier function [88]. DSS-induced colitis mouse model study demonstrated that propionate reduces inflammation and oxidative stress as well as reinforces the barrier function [89]. SCFAs of different concentrations generated from the fermentation of different dietary fibers showed protective as well as reinforcing effects on epithelial barrier function [90].

Probiotics can improve the intestinal barrier function and repair the damages in intestinal epithelial cells [91,92]. Improving the intestinal barrier could be a possible strategy to restrict the cancer invasion and metastatic process by targeting matrix metalloproteinases (MMPs) and plasminogen activators, which degrade the Extracellular matrix (ECM) that comprises the integrity of endothelial basement membrane and mesenchymal collagen [93]. Expression of Matrixmetalloproteinase-9 (MMP-9) decreased and zona occludens (ZO)-1, a tight junction protein, increased in HCT116 cells when treated with cell-free supernatant (CFS). Macromolecules obtained from the size fractionation process of CFS of *Lactobacillus casei* (ATCC 334), and *Lactobacillus rhamnosus* GG (ATCC 53103) (LGG), decreased the cell invasion capacity in vitro. The inhibitory effect was supposed to be driven by macromolecules that have a molecular weight of 50–100 kDa/>100 kDa [44]. (Table 1 compiles some evidence where probiotics improved intestinal barrier function.).

### 2.5. Production of Anticarcinogenic Compounds

Anticarcinogens are bioactive compounds that can inhibit the process of the development of cancer or can neutralize different carcinogenic compounds. The probiotics can restrict carcinogenic events by secretion of such compounds. Apart from the SCFAs, the probiotic microorganisms also produce conjugated linoleic acids and some bioactive compounds, which exert cytotoxic effects on cancerous cells. These bioactive compounds are often extracellular in nature and can be extracted to test their effect on colon cancer cell lines. Some of them show better tumor-suppressive effects than some of the approved chemotherapeutic drugs for colorectal cancer treatment [45].

The anticarcinogenic compound ferrichrome is produced by the *Lactobacillus casei* ATCC334 strain, which showed tumor-suppressive effects on Caco-2/bbe(clone of Caco-2, human colorectal adenocarcinoma), SKCO-1, and SW620 (Human colorectal adenocarcinoma). The anticarcinogenic effect is observed to be driven by the activation of c-jun N-terminal kinase (JNK) [45]. Moreover, ferrichrome showed a better tumor-suppressive effect on HCT116, HT-29, and SW-480 cancerous cell lines when compared to chemotherapeutic drugs such as 5-FU(Fluorouracil) and cisplatin. Interestingly, the combination of ferrichrome and 5-FU showed a synergistic antitumor effect than 5-FU alone, which might be driven by the upregulation of DDIT3 (DNA Damage Inducible Transcript 3). DDIT3 is a pro-apoptotic transcription factor that can induce apoptosis through mitochondria-Dependent or death ligand-receptor pathway by regulating BAK, BAX, BCL2, BCL-X, Fas, TNF, and TRAIL [46]. Another study with the cell-free supernatant (CFS) and extracted active metabolites (primarily composed of organic acids and proteins) from CFS of *L. rhamnosus* MD 14 showed antigenotoxic effect against cancer-inducing genotoxic compounds and showed a cytotoxic effect in Caco-2 and HT-29 cells, which is marked by an arrest of the cell cycle in G_0_/G1 phase [47]. Similarly, CFS of *Lactobacillus casei* (LC-WT, ATCC 334, wild type) and *L. casei* (LC-CLA) (Conjugated linoleic acid (CLA) overexpressing) containing conjugated linoleic acid induces cellular apoptosis in HCT116 cells. The apoptosis is induced by downregulation of tumor growth genes (cyclin-dependent kinases)-1/2/6, PLK1 (serine/threonine-protein kinase/polo-like kinase 1), and SKP (S-phase kinase-associated protein), on the other hand, induce upregulation of pro-apoptotic genes such as JUN (Jun Proto-Oncogene), BBC3 (BCL2 Binding Component), and DDIT3. The cell-free culture supernatant was also observed to induce the secretion of anti-inflammatory cytokines (IL-10 and TGF-β) and pro-inflammatory cytokines (IL-1β, INF-γ, and TNF-α) [48]. A newly isolated compound plantarone from the culture filtrate of probiotic *Lactobacillus plantarum* H24, having a structure of 5,7-peroxide of kojic acid, showed a cytotoxic effect on Caco-2 cells of up to 60.72 ± 3.55% with an IC_50_ value of 50.2 ± 0.28 µM [49].In another investigation, *Lactobacillus johnsonii* BCRC17010 shown good adhesion properties to HT-29 cells and can induce cell apoptosis by intrinsic BAX/BCL-2 pathway whereas *L. reuteri* BCRC14625 exerts harmful effect to HT-29 cell membrane by secretion of lactate dehydrogenase (LDH) [43]. An experimental outcome suggested that *Lactobacillus pentosus* B281 and *Lactobacillus plantarum* B282 produce thermostable bioactive compounds, which exert an antiproliferative effect on the Caco-2 cell line [31]. (Table 1 compiles evidence where probiotics produce anticarcinogenic and their effects on cell lines in vitro).

### 2.6. Degradation of Carcinogenic Compounds

Metabolic enzymes produced from intestinal microbiota often bio transform different precursor compounds into carcinogenic compounds. The cells of the colon and rectum are often exposed to different carcinogenic compounds. These are mainly mutagens and pro-mutagens such as benzo(a)pyrene, sodium azide, N-methyl-N9-nitro-N-nitrosoguanidine (MNNG), IQ, aflatoxin B1 (AFLB1), and 3-amino-I,4-dimethyl-5H-pyrido (4,3-b) indole (TrpP-1) [61]. Intestinal microbes can degrade and alter (detoxify) the xenobiotics from dietary components such as phytochemicals, lipids, and proteins, medication components (pharmaceuticals), etc., producing various enzymes. Different carcinogenic factors found in food are found to be responsible for the development of colon cancer [94]. It is also evident that the consumption of red meat has a significant contribution to the development of colorectal cancer. N-nitroso compound (NOC), heterocyclic amines (HCAs), and polycyclic aromatic hydrocarbons (PAHs) are often generated by meat and are potent mutagens [95]. Modulating the enzyme expression of different intestinal microbes, thereby generation of mutagenic and carcinogenic compounds can be controlled indirectly.

Probiotics can directly inactivate carcinogens by binding to the carcinogens or by decreasing the activity of the compound. Different probiotics strains and Lactic acid bacteria (LAB) can remove carcinogen benzo(a)pyrene, a PAH from in vivo experimental models as well as from food. The potential mechanism involves the physical binding of carcinogens to the peptidoglycan of the bacterial cell wall or by the active metabolism of the compound [96]. A non-human origin probiotic strain of Lactobacillus *plantarum* CM4 can degrade nitrosamine and can remove mutagens such as 2-amino-1-methyl-6-phenylimidazo [4,5-b] pyridine (PhIP) and 2-amino-3-methylimidazo[4,5-f] quinoline (IQ) [50]. Another probiotic, *Lactobacillus casei* DN 114001, has shown in vitro evidence of removal of mutagens such as IQ, MelQx, and PhIP when cultured in MRS broth. The metabolism or adsorption efficiency of strain depends on the type of medium, growth of the cell, and incubation time [51]. *Lactobacillus plantarum* 301102, a mutant strain, produces exopolysaccharides (EPS), which can bind to heterocyclic amines and can inactivate the mutagen where the binding of mutagen is pH-dependent [52]. Another study showed probiotic yeasts *Kluyveromyces lactis* VIT-MN02 isolated from millet root, *Lipomycesstarkeyi* VIT-MN03, and *Saccharomycopsisfibuligera* VIT-MN04 isolated from goat intestine act as antigenotoxic against 4-NQO and MNNG, anticancer against Caco-2 cell lines, and antimutagenic against sodium azide (SA), pro-mutagen benzo-amino pyrene B[a]P and acridine orange (AO) [53]. (Table 1 compiles a list for degradation of the mutagenic compounds by probiotics).

## 3. Extension of Evidence of Prophylactic Action of Probiotics in CRC Obtained from Animal Studies

The results from in vivo studies on different animal models established strong evidence for the use of probiotics or probiotics combined with prebiotics (synbiotic) for the prevention and treatment of colorectal cancer. Synbiotics are a combination of probiotics and prebiotics where prebiotics stimulate the selective growth of specific microbes. A systematic review on preclinical trials deciphering the significance of probiotics and synbiotics on colorectal carcinogenesis yields around 33–34 in vivo studies till the year 2018. Experimental outcomes suggested that the used probiotics area potential alternative to the conventional methods used for the management of CRC. The review suggested that most of the studies were performed using either rats or mice as a model organism, and the tumor or preneoplastic lesions were induced mostly by 1,2 Dimethyl Hydrazine (DMH), rarely in some cases by Azoxymethane (AOM) and inoculation of cancerous CT26 cells. The probiotics tested commonly belonged to *Lactobacillus* and *Bifidobacterium* genera but also included genera such as *Streptococcus*, *Clostridium*, *Bacillus*, *Lactococcus*, an established cocktail VSL#3, and fungi such as *Saccharomyces boulardii*. The authors also found that *Lactobacillus acidophilus* and *Lactobacillus plantarum* are mostly used probiotic species [97,98].

Many pieces of evidence suggest that probiotics can decrease the incidence of tumors, often counteract the effect of mutagens, and can be used as a prophylactic treatment for CRC. The antitumor immune response of two LAB strains, *Lactobacillus plantarum* A and *Lactobacillus rhamnosus* b, were investigated in a subcutaneous and orthotopic model of CT26 murine adenocarcinoma cells in BALB/c mice. In a positive outcome *Lactobacillus plantarum*, reduced tumor growth by enhancing the innate immune response in a series of events such as dendritic cell maturation for polarization of Th1 response, CD8+, and NK cells migration and resulted in reduced tumor growth and prolonged survival of the animal. On the contrary, *Lactobacillus rhamnosus* failed to reiterate those outcomes [99]. Z.-F. Chen et al. showed the knowledge about the molecular mechanism involved in restricting inflammation and elevating immune homeostasis by *C. butyricum* and *B. Subtilis* in a DMH-induced CRC model using male C57BL/6 mice. The proliferation of cancer cells was downgrade following cell cycle arrest and promoting apoptotic mechanisms [59]. Again, in a 1,2-dimethylhydrazine (DMH)-induced rat model, independent researchers have found out that Lactobacillus strains effective as a prophylactic measure. The antigenotoxic effect of probiotics curd (mixture of both Lactobacillus and Lactococcus cultures) exhibited a protective effect in colonic cells of the animal and showed significantly less DNA damage in the animals consuming probiotics curd with DMH injection [100]. In one more similar study, the effects of probiotic Dahi (*Lactobacillus acidophilus* LaVK2 and *Bifidobacterium bifidum* BbVK3 individually or together with piroxicam (PXC)) was tested on biomarkers of CRC such as preneoplastic lesions. The results suggested anti-neoplastic and antiproliferative action of the probiotics Dahi and could help prevent initiation and progression of CRC in the DMH-treated male Wistar rats [101]. *Lactobacillus salivarius* Ren (Ren) prevented colon cancer by modulating the gut microflora in the DMH-injected rat. Ren could alter the DMH-induced adverse effects by reversal of the gut microbiota close to the healthy state in the rat, and the incidence of cancer was reduced from 87.5% to 25% [102].

The effects of a consortium of probiotics and the use of synbiotic have shown tremendous potential in CRC models in vitro. Some evidence shows the effect of probiotics along with prebiotics can modulate the immune system and can promote apoptosis in tumor cells in vivo also. Germinated Brown rice (GBR) and fermented GBR by *Lactobacillus acidophilus* reduce the occurrence of Aberrant crypt foci (ACF) and different cytokines such as TNF-α, IL-6, and IL-1β along with increased expression of pro-apoptotic markers such as cleaved caspase-3 and decreased expression of antiapoptotic markers such as Bcl-2, in male F344 rats treated with DMH/DSS [103]. Another synbiotic combination of Germinated Brown Rice (GBR) and *Lactobacillus acidophilus* LA5, *Bifidobacterium animalis* subsp. *Lactis* BB-12, inhibited the formation of mucin depleted foci (MDF) in the middle colon and ACF-producing sialomucin (SIM-ACF), marked by increased expression of pro-apoptotic markers such as P53, caspase-3 and decreased expression of antiapoptotic Bcl-2, in 1,2-dimethylhydrazine (DMH) and dextran sulfate sodium (DSS)-treated male F344 rats [104]. A decreased occurrence of ACF, SIM-ACF, and MDF was seen in DMH/DSS-treated male F344 rats when supplemented with a combination of Djulis (grain containing dietary fiber with prebiotic attributes) and *Lactobacillus acidophilus* LA-5, which is driven by the downregulation of proliferation and inflammation-related proteins such as PCNA and COX-2 and modulation of apoptotic pathway-related proteins such as Bcl-2, BAX, and caspase-3 [105].

It is well known that a moderate level of Reactive Oxygen Species (ROS) can exert cellular damage and cause mutations in DNA, leading to the development of cancer. At the same time, it is evident that the level of ROS is found to be higher in colorectal cancer cells than in other normal tissues [106]. Antioxidant enzymes, such as superoxide dismutase (SOD), catalase, thioredoxin reductase, glutathione reductase, glutathione peroxidases, and glutathione S-transferases, are also known as scavengers of free ROS have a crucial role in balancing the redox system in the cells and tissues [107]. In vivo studies have recorded evidence that shows that probiotics can reduce the chances of damage and mutation caused by ROS and free radicals. In vivo evidence also suggests the antioxidant activity of probiotics. It is found that DMH decreases the enzymes Glutathione (GSH), superoxide dismutase (SOD), catalase (CAT), glutathione reductase (GR), glutathione peroxidase (GPx), glutathione-S-transferase (GST), and upon administration of probiotics, the effect reversed, leading to an increase in the concentration of antioxidant enzymes [108].

Probiotics can control the oncogenic events via miRNA-based molecular pathways. It is found that Probiotic *Bifidobacterium bifidum* and *Lactobacillus acidophilus* when administered to AOM induced colorectal male BALB/c mice model inhibited the expression of oncomirs such as miR-135b, miR-155 and increased expression of miR-26b, miR-18a accompanied by regulating the expressions of KRAS (oncogene), and tumor suppressor genes such as PU.1, APC, PTEN [109]. A commercial probiotic that contains *Lactobacillus acidophilus* (NCFM^R^), *Lactobacillus paracasei* (Lpc 37^TM^), *Bifidobacterium lactis* (Bi-04^TM^), *Bifidobacterium lactis* (Bi-07^TM^), and *Bifidobacterium bifidum* (Bb-02^TM^), when administered alone or in combination with 5-FU, in DMH-induced colorectal male Fischer F344 rats, have decreased the number of formations of ACF and malignant neoplastic lesions [110], suggesting the use of probiotics as an adjuvant with other chemotherapeutic drugs.

The intestinal microflora is effective in modulating host metabolism by producing a plethora of enzymes that can in another way affect both microbes and host mutually. There are preliminarily three types of metabolic reactions carried out by intestinal microflora, which involve hydrolysis, reduction, and dihydroxylation. The diverse and large microbial population of the intestine uses substances that enter the intestine as a substrate and convert them to different products by using different enzymes such as β -glucosidase, β-galactosidase, β -glucuronidase, azoreductase, and nitroreductase. The products generated from these reactions are often carcinogenic in nature [111]. Alteration of intestinal metabolism and decrease in the production of enzymes such as β-glucuronidase, β-glucosidase, and nitroreductase is also carried out by some probiotics. This is demonstrated by a decrease in the number of fecal enzymes upon administration of probiotics in animal models [16,112,113,114,115,116,117].

**Figure 2:**
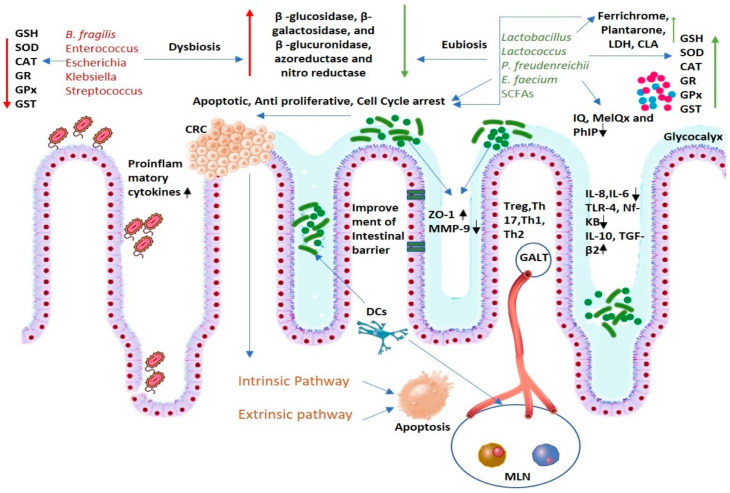
Different mechanisms of probiotics action in the prevention and treatment of colorectal cancer. Tumor induction, proliferation, and metastasis are often accelerated by dysbiosis conditions, which are associated with an increase in inflammation, production of enzymes that can convert different precursor compounds into carcinogens, and loss of the integrity of intestinal barrier function. Probiotics and their products, such as Short-chain fatty acids (SCFAs), exert an apoptotic, antiproliferative, and cell cycle arrest effect on cancerous cells induced by extrinsic and intrinsic apoptotic pathways. They create homeostasis and eubiotic conditions by competing with harmful pathogens and increasing tolerance of immune cells to commensal microbes by regulating the immune systems, thereby regulating the secretion of different pro-inflammatory cytokines such as IL-8 and IL-6 and anti-inflammatory cytokines such as IL-10 and TGF-β2. They secret anticancer compounds such as ferricrome, plantarone, conjugated linoleic acid (CLA), lactate dehydrogenase (LDH) exerts an anticancer effect on tumor cells. They improve the intestinal barrier by increasing the expression of zona occludens (ZO)-1 and decreasing the expression of Matrix metalloproteinase-9 (MMP-9). They increase the expression of Reactive oxygen species scavenging enzymes such as glutathione (GSH), superoxide dismutase (SOD), catalase (CAT), glutathione reductase (GR), glutathione peroxidase (GPx), glutathione-S-transferase (GST). They can stimulate the differentiation of T-cells into Treg, Th1, Th2, and Th17, which regulate inflammation and creates homeostatic conditions. They can greatly alter the metabolism pattern of harmful microbes and reduce the number of enzymes, such as β-glucuronidase, β-glucosidase, and nitroreductase, thereby decreasing the production of carcinogenic compounds. They can directly bind and remove carcinogenic compounds such as 2-amino-1-methyl-6-phenylimidazo[4,5-b] pyridine (PhIP), 2-amino-3-methylimidazo[4,5-f] quinoline (IQ), 2-amino-3,8-dimethylimidazo[4,5-f] quinoxaline (MeIQx). (MLN: Mesenteric Lymph node; GALT: Gut-associated Lymphoid Tissues).

Different mechanisms adopted by probiotics for the management of colorectal cancer are depicted in Figure 2.

## 4. Status of Clinical Outcomes

Several clinical trials yield results that provide strong evidence for the use of probiotics as a supplement treatment strategy. The outcomes of such clinical trials mainly (but not limited to) include an increase in beneficial bacterial diversity, reduction of Fusobacterium and Peptostreptococcus species, improvement in gut functionality, decrease in transmucosal permeability, improvement in epithelial barrier function, decrease in proliferation of tumor cells, and a decrease in symptoms related to irritable bowel disease [118,119,120,121,122,123]. A randomized trial conducted on a total of 398 subjects containing both men and women has shown that there is a lower rate of occurrence of moderate and higher atypia graded tumors when administered with the preparation of *Lactobacillus casei*. The above outcome suggests the prophylactic implication of probiotics in CRC [124]. Additionally, perioperative administration of probiotics or synbiotics reduced the postoperative complications as well as reduced the symptoms associated with the gastrointestinal environment. The outcomes include a decrease in the length of hospital stays, lower occurrence of septicemia, lower incidence of postoperative infections, decreased incidence of diarrhea, a faster recovery rate, and a reduced rate of postoperative antibiotics use [125]. It is also seen that an improvement in overall quality of life and alleviation of side effects generated by chemotherapy when the subjects were administered with a combination of strain-specific probiotic (microbial cell preparation) and omega-3 fatty acid [126].

Although the outcomes from clinical trials are highly encouraging still the number of trials relevant to this field is very limiting. Randomized clinical trials with a larger sample size, proper randomization process, proper analysis, and validation must be conducted to create more comprehensive and concrete evidence to support the theory.

## 5. Conclusions

Consumption of probiotics to get health benefits is becoming a common practice. The uses of probiotics or fermented foods, such as yogurt, which contain LAB, were tested since 1980, given their effect on IBD and CRC. Although there are many in vitro and in vivo evidence that supports the concept of the use of probiotics for colorectal cancer management, the demonstration of this concept in clinical trials is still in the preliminary stage. The mechanisms of action of probiotics are numerous to ameliorate the progression of CRC. Considering their effectiveness in managing colorectal cancer, we should prioritize establishing more concrete mechanisms and identify key targets to exploit probiotics and increase the efficiency of the treatment. The efficiency of probiotics is again a matter of strain-specific nature, and the results from one organism can’t be extrapolated to others. In view of the fact that the number of colorectal cancer patients is increasing considerably, isolation, screening, and selection of novel probiotics/probiotic strains is desirable. Mostly all the suggested criteria for the prevention of CRC work constitutively, so a testing consortium of probiotics/ Synbiotic/metabiotics and their effects through in-vitro, in vivo, and most importantly by clinical trials is the need of the hour. Suggested by few researchers, one bottleneck in using probiotics for cancer therapy is the detrimental effects bore by the host due to live bacterial treatment that results in bacteremia; therefore, the use of metabiotic is recommended, which are the active components of probiotics metabolites.

In many cases, it is found that when probiotics are administered with other chemotherapeutic drugs, they increase the efficiency of treatment by several folds; therefore, the use of probiotics as an adjuvant could be a potential area of research. As probiotics are reported to reduce oxidative stress by scavenging the reactive oxygen and nitrogen species (RONS) molecules through the production of antioxidant enzymes and modulation (such as metal ion chelation, regulation of signaling pathways, alteration of intestinal microbiota, etc.), they can be used alongside chemotherapy wherein a large amount of ROS are generated. Above all, it is evident that probiotics can modulate our immune system by influencing the polarization of immune cells and controlling the secretion of cytokines. This indicates the use of probiotics in treatment strategies, especially in targeted immunotherapy, to increase the chances of successful treatment. On a concluding note, probiotics (mainly Lactobacillus and Bifidobacterium) are generally regarded as safe (GRAS) by the US Food and Drug Administration and could be used without any adverse effect on the host. It has been shown that the intra-tumor immune composition could be a major determinant of clinical outcomes in CRC. Hence, Immunoscore has been proposed as a new component of TNM-Immune classification in cancer [127]. The quality and the density of immune infiltrates in the tumor microenvironment are affected by multiple factors, including gut microbiota. As probiotics act as mucosal and systemic immune modulators, based on the Immunoscore of CRC patients, personalized probiotics could be adopted for cancer therapy.

## Figures and Tables

**Table 1:** Different probiotic strains and their effect on different cell lines in vitro.

Probiotic Strains (Bacteria/Yeast)	Cell Culture	Effect	References
*Propionibacterium acidipropionici* *Propionibacterium freudenreichii*	HT29 Caco-2	Apoptosis Caspase 3 ↑ Propionate and acetate ↑	[20]
*Lactobacillus rhamnosus* GG (LGG) *Bifidobacterium lactis* Bb12	LT97 HT29	Cell cycle arrest Apoptosis check on G0/G1 p21 and WNT2B * Butyrate, acetate, and propionate ↑	[21]
*Pediococcuspentosaceus* FP3 *Lactobacillus salivarius* FP25 *L. salivarius* FP35 *Enterococcus faecium* FP51	Caco-2	Antiproliferative Adherence Butyric and propionic acids ↑	[22]
*Propionibacterium freudenreichii*	HT29	Apoptosis TRAIL-R1/DR4, TRAIL-R2/DR5 ↑ Caspase-3, -8, -9 Bcl-2 ↓ Propionate and acetate ↑	[23]
*Lactobacillus casei* ATCC 393	CT26 HT29	Antiproliferative Pro-apoptotic cyclin D1 and BIRC5a ↓ TRAIL ↑	[24]
*Lactobacillus plantarum* A7 (Heat killed) *Lactobacillus rhamnosus* GG (heat Killed)	Caco-2 HT-29	Antiproliferative organic acid ↑	[25]
*Enterococcus faecium* RM11 *Lactobacillus fermentum* RM28	Caco-2	Antiproliferative	[27]
*Bacillus polyfermenticus*	HT-29 DLD-1 Caco-2	Growth inhibition Antiproliferative ErbB2 and ErbB3 ↓	[30]
*Lactobacillus acidophilus* (ATCC 4356) (CS and CE) *Lactobacillus casei* (ATCC 39392) (CS and CE)	Caco-2	Antiproliferative Apoptotic Necrosis Antimetastatic	[33]
*Lactobacillus acidophilus* LA102 (CE) *Lactobacillus casei* LC232 (CE)	Caco-2 HRT-18	Antiproliferative Cytotoxic activity	[32]
*Lactobacillus casei* UT1 (CS)	HCT116	Pro-apoptotic	[26]
*Streptococcus thermophilus* (19258)	HCT116 HT29 Caco-2	Apoptosis, Antiproliferation cell cycle arrest Anti-Warburg effect Energy Homeostasis * Galactose ↑	[38]
*Saccharomyces cerevisiae* PTCC 5052 (HI)	SW480	Apoptosis Akt/NF-κB signaling * BAX ↑ Cleaved caspase-3 and caspase-9 ↑ Bcl-XL ↓ Pro-caspase 3, 9 ↓ p-Akt1 ↓ Rel A ↓	[39]
*Lactobacillus johnsonii* BCRC17010 *L. reuteri* BCRC14625	HT-29	Apoptosis Cytotoxic BAX ↑ Bcl-2 ↓ Lactate dehydrogenase (LDH) ↑	[43]
*Lactobacillus pentosus* B281 (CM) *Lactobacillus plantarum* B282 (CM)	Caco-2	Antiproliferative Cell cycle arrest Cyclins A, B1, B2 and E ↓	[31]
*Lactobacillus acidophilus* 606 (Cell bound EPS)	HT29	Autophagy Beclin-1 ↑ GRP78 ↑ Bcl-2 and Bak *	[34]
*Lactobacillus plantarum-12* (Extracted EPS)	HT-29	Antiproliferative Proapoptotic Proliferating cell nuclear antigen (PCNA) ↑ Bax ↑ Cyt C ↑ Caspase-3, -8, -9 ↑ Bcl-2 ↓ Reactive Oxygen Species (ROS) ↓	[35]
*Lactobacillus casei* (ATCC 334) *Lactobacillus rhamnosus* GG (ATCC 53103) (LGG)	HCT-116	Matrixmetalloproteinase-9 (MMP-9) ↓ zona occludens (ZO)-1 ↑	[44]
*Lactobacillus casei* ATCC334	Caco-2/bbe SKCO-1 SW620	Production Ferrichrome Activate N-terminal kinase (JNK) Antitumor/Tumor suppressive effect	[45]
*Lactobacillus casei* ATCC334	HCT116 HT29 SW480	Tumor suppressive DDIT3 ↑	[46]
*Lactobacillus rhamnosus* MD 14 (CFS)	Caco-2 HT-29	Antigenotoxicity against β-galactosidase Cytotoxic effect Cell cycle arrest	[47]
*Lactobacillus casei* (LC-WT, ATCC 334) (Wild type) *L. casei* (LC-CLA) (Conjugated linoleic acid (CLA) overexpressing) ^#^	HCT-116	CDK1/2/6, PLK1, and SKP2 ↓ inflammatory cytokines ↓ JUN, BBC3, and DDIT3 ↑ Anti-inflammatory cytokines ↑	[48]
*Lactobacillus plantarum* H24	Caco-2	Production of Plantarone Cytotoxic effect	[49]
*Lactobacillus johnsonii* BCRC17010 *L. reuteri* BCRC14625	HT-29	Apoptosis BAX ↑/BCL-2 ↓ Production of lactate dehydrogenase (LDH) Harm on cell membrane	[43]
*Lactobacillus pentosus* B281 *Lactobacillus plantarum* B282	Caco-2	Production of Bioactive compound Antiproliferative effect	[31]
*Lactobacillus plantarum* CM4	-	Degrade nitrosamine Remove mutagens such as PhIP and IQ	[50]
*Lactobacillus casei* DN 114001	-	Remove mutagens IQ, MelQx and PhIP	[51]
*Lactobacillus plantarum* 301102 ^#^	-	Produce exopolysaccharides Inactivate mutagens heterocyclic amines	[52]
*Kluyveromyces lactis* VIT-MN02 *Lipomycesstarkeyi* VIT-MN03 *Saccharomycopsisfibuligera* VIT-MN04	Caco-2	Antigenotoxic against 4-NQO and MNNG Anticancer effect Antimutagenic against SA, B[a]P, AO	[53]
*Lactobacillus acidophilus* CL1285 *Lactobacillus casei* LBC80R + 5-FU	LS513	Sensitivity towards drugs ↑ Apoptotic effect Caspase-3 ↑ p21 ↓	[41]
*Lactobacillus plantarum* (CCARM 0067) (CS)	HT-29 HCT-116	Sensitivity towards drugs ↑ caspase 3 ↑ Wnt/β-catenin ↓ CD44, CD133, CD166, ALDH1 ↓	[42]
live *Lactobacillus Rhamnosus* GG (LGG)/UV-inactivated LGG	Caco-2	IL-8 ↓ Restricts NF-κB translocation	[54]
*Lactococcus lactis* NK34	DLD-1, HT-29 and LoVo RAW 264.7	Antiproliferative effect Immunomodulation Pro-inflammatory cytokine ↓ Nitric oxide ↓	[55]
*Bacillus polyfermenticus* KU3	LoVo HT-29	Antiproliferative Effect IL-10, TGF-β2, COX-2, TNF-α ↓ Nitric oxide ↓	[56]
*Bifidobacterium longum*, *Bifidobacterium bifidum*, *Lactobacillus acidophilus*, *Lactobacillus plantarum* + Prebiotics	CT26	Antiproliferative effect Antimetastatic effect CD8+ T cell ↑	[57]
*Bifidobacterium adolescentis* SPM0212 (CFS)	HT-29 SW 480 Caco-2 RAW-264.7	Growth inhibition Production of TNF-α	[58]
*Clostridium butyricum* (ATCC 19398) (CM) *Bacillus subtilis* (ATCC 23857) (CM)	HCT116 SW1116	Apoptotic Cell cycle arrest TLR4 and NF-κB ↓ Th17 * p-ERK ↓ P21 ↑	[59]

CE: Cell Extract; CFS: Cell Free Supernatant; CS: Culture Supernatant; CM: Conditioned Medium; HK: Heat Killed; HI: Heat Inactivated; *: Modulation; ↓: Down regulation/Reduced expression; ↑: Upregulation/Increased expression; EPS: Exopolysaccharides; ^#^: Mutant strain.
